# Development of High Cell Density Cultivation Strategies for Improved Medium Chain Length Polyhydroxyalkanoate Productivity Using *Pseudomonas putida* LS46

**DOI:** 10.3390/bioengineering6040089

**Published:** 2019-09-26

**Authors:** Warren Blunt, Christopher Dartiailh, Richard Sparling, Daniel J. Gapes, David B. Levin, Nazim Cicek

**Affiliations:** 1 Department of Biosystems Engineering, University of Manitoba, Winnipeg, MB R3T 5V6, Canada; umdartia@myumanitoba.ca (C.D.); david.levin@umanitoba.ca (D.B.L.); Nazim.Cicek@umanitoba.ca (N.C.); 2 Department of Microbiology, University of Manitoba, Winnipeg, MB R3T 2N2, Canada; Richard.Sparling@umanitoba.ca; 3 Scion Research, Te Papa Tipu Innovation Park, Sala Street, Private Bag 3020, Rotorua 3046, New Zealand; Daniel.Gapes@scionresearch.com

**Keywords:** polyhydroxyalkanoates, fed-batch, productivity, *Pseudomonas*, bioreactor, microaerophilic

## Abstract

High cell density (HCD) fed-batch cultures are widely perceived as a requisite for high-productivity polyhydroxyalkanoate (PHA) cultivation processes. In this work, a reactive pulse feed strategy (based on real-time CO_2_ or dissolved oxygen (DO) measurements as feedback variables) was used to control an oxygen-limited fed-batch process for improved productivity of medium chain length (mcl-) PHAs synthesized by *Pseudomonas putida* LS46. Despite the onset of oxygen limitation half-way through the process (14 h post inoculation), 28.8 ± 3.9 g L^−1^ total biomass (with PHA content up to 61 ± 8% cell dry mass) was reliably achieved within 27 h using octanoic acid as the carbon source in a bench-scale (7 L) bioreactor operated under atmospheric conditions. This resulted in a final volumetric productivity of 0.66 ± 0.14 g L^−1^ h^−1^. Delivering carbon to the bioreactor as a continuous drip feed process (a proactive feeding strategy compared to pulse feeding) made little difference on the final volumetric productivity of 0.60 ± 0.04 g L^−1^ h^−1^. However, the drip feed strategy favored production of non-PHA residual biomass during the growth phase, while pulse feeding favored a higher rate of mcl-PHA synthesis and yield during the storage phase. Overall, it was shown that the inherent O_2_-limitation brought about by HCD cultures can be used as a simple and effective control strategy for mcl-PHA synthesis from fatty acids. Furthermore, the pulse feed strategy appears to be a relatively easy and reliable method for rapid optimization of fed-batch processes, particularly when using toxic substrates like octanoic acid.

## 1. Introduction

The detrimental effects from accumulation of plastic waste in natural environments call for change at both the regulatory and behavioral levels [1]. Most (60–95%) ocean plastic has been classified as single-use in origin [2]; consequently, these single-use items are being increasingly banned by governments globally [3]. Alternative materials must be developed to offset the reduction in single-use items, and biodegradable polymers may be part of that solution. 

Microbial polyhydroxyalkanoates (PHAs) are a promising class of biopolymers that are both renewable (bio-based) and biodegradable. PHAs are synthesized by a variety of microbial species, typically in environments not suitable for growth, but where excess carbon is present. There are two main classes of PHAs defined by the carbon chain-length of the monomer subunits: (1) short chain length (scl-) PHAs, which consist of C3 to C5 monomer subunits; and (2) medium chain-length (mcl-) PHAs, which consist of C6 to C18 monomer subunits [4]. Some of these PHA polymers have properties comparable to conventional petrochemical plastics, including polyethylene and polypropylene [5], and may indeed be suitable alternatives. 

Despite promise, the cost of production has limited commercial-scale production of PHAs, particularly for the mcl-PHAs [6,7]. There are a number of economic drivers for this, including the relatively recent drop in oil prices arising from increased exploitation of shale oil [8]. Production costs for PHA have been estimated to be as much as fifteen-fold higher than the petrochemical counterparts (polyethylene, polypropylene) they are intended to replace [9]. Development of productive and cost-effective bioreactor cultivation processes is an important aspect of improving the economic viability and lessening the environmental impacts of PHA production [10,11,12]. 

The overall volumetric productivity of any bioprocess is a crucial performance metric and is typically around 2 g L^−1^ h^−1^ for commercial production of bulk bio-products [13]. In PHA production, high cell density (HCD) cultures are widely seen as the best cultivation strategy to reduce costs by achieving high bioreactor productivities [14]. Several studies investigating HCD fed-batch cultivation strategies have obtained productivities of around 2 g L^−1^ h^−1^ in mcl-PHA research [15,16,17,18]. On the other hand, cell densities in excess of 200 g L^−1^ and productivities of 5.13 g L^−1^ h^−1^ have been reported for scl-PHA production operations [19,20,21]. While these results are indeed impressive, many of these studies were performed at bench-scale and used an aeration medium with enriched oxygen content, which can add significant costs in a large-scale setting. A recent survey of scale-up operations in PHA production pointed out that productivities reported for scaled-up operations have, for some time, been lagging behind those achieved in the lab [22]. This discrepancy is at least partly attributable to poor mass transfer characteristics within large-scale bioreactors, especially when it comes to maintaining dissolved oxygen (DO) in aerobic bioprocesses [23,24]. 

In this context, practical constraints prevent the solution from being as simple as dissipating more energy for mixing, aeration, and purifying oxygen for large-scale bioreactors. A life cycle analysis of scl-PHA synthesized from whey indicated that mechanical energy required for mixing during the fermentation process was one of the main factors contributing to the high environmental impact (and cost) of PHA production—even without mention of aeration supplementation with purified oxygen [25]. This highlights the need to develop processes at lab-scale that are representative of the environment that might be encountered in pilot- or industrial-scale bioreactors. 

Previously, we have shown that in the production of mcl-PHAs from medium and long chain fatty acids, imposing O_2_ limitation on the culture results in a significant redirection of carbon flux toward PHA synthesis [26,27]. However, the aforementioned studies used batch or simple fed-batch strategies as proof-of-concept, and overall productivities were relatively low. Improving the cell density of the cultivation using an appropriate feeding strategy under oxygen limited conditions could further improve overall productivity. Eventual scaled-up mcl-PHA cultivation processes may also benefit from an understanding of oxygen-limited metabolism brought on by a HCD environment as well as reduced energy consumption (for less rigorous mixing and aeration) resulting from oxygen-limited product synthesis. 

In addition to maintaining an adequate supply of oxygen, there are several challenges that arise from HCD cultures. Monitoring and control of these processes is difficult because of the lack of real-time measurements of key process variables, such as substrate uptake [28,29]. This is particularly problematic when the substrate exhibits toxicity, and accumulation of substrate can have deleterious consequences [30]. Previously it was found that a fine threshold exists between carbon-limited growth and substrate-induced inhibition when feeding octanoic acid at a predetermined exponential rate (i.e., a proactive feeding approach) [26]. In those experiments, it was observed that carbon limitation was indicated by a rapid and sharp decrease in the off-gas CO_2_ concentration, and subsequent rise in the DO signal, when feeding was briefly paused. Thus, a possible solution to the toxicity issue may be to use a reactive feeding approach; that is, to feed sub-inhibitory amounts of carbon when needed, as indicated by real-time signals, rather than feeding at a predetermined rate. 

Similar strategies have been used in the past. These have employed process indicators such as pH, dissolved oxygen (DO), and CO_2_ production [17,29,31,32]. As pointed out by Riesenberg and Guthke [28], these strategies depend on carbon-limited growth, and thus may limit growth and product formation rates. While multiple studies have used predetermined feeding rates rather successfully [18,33,34], a reactive approach may be preferable during initial process optimization. 

The objectives of this work, therefore, were: 1) to apply O_2_-limited mcl-PHA synthesis from fatty acids to HCD cultures of *Pseudomonas putida* LS46; and 2) to evaluate the carbon flux for different feeding strategies used to obtain HCD cultures. This was done with the motivation of the development and evaluation of simple, effective, and readily scalable feeding strategies for improving mcl-PHA productivity, in addition to understanding the limits of oxygen limitation on mcl-PHA cultivations in a HCD environment. 

## 2. Materials and Methods

### 2.1. Micro-Organism, Medium, and Substrates

The strain used in this study was *Pseudomonas putida* LS46 [35]. Strain culturing and maintenance procedures were as specified previously [27]. Ramsay’s minimal medium [36] was used in all studies. However, the initial concentrations of (NH_4_)_2_SO_4_, MgSO_4_, CaCl_2_·2H_2_O, and trace element solution were increased to 2 g L^−1^, 0.2 g L^−1^, 20 mg L^−1^, and 2 mL L^−1^, respectively. The MgSO_4_, CaCl_2_·2H_2_O, ferric ammonium citrate, and trace element solution were filter-sterilized through a 0.2 μm filter after autoclaving. Octanoic acid was used as the substrate with an initial concentration of 20 mM and was added through a sterile 0.2 μm filter after autoclaving. 

### 2.2. Reactor Setup and Operation 

A 7 L autoclavable glass reactor was used (Applikon, Foster City, CA, USA) with an initial working volume of 3 L. The reactor setup, sensor calibration, and sterilization procedures were identical to those previously described [27]. Experiments were initiated with the addition of a 5% (vol vol^−1^) inoculum from an overnight culture of Ramsay’s medium with 20 mM octanoic acid (9.5 mL into 3 L) as the carbon source. The medium pH was maintained at 6.5, which was controlled through the addition of 4 M NaOH via automated peristaltic pumps. The intended DO set point was 40% (of air saturation at 30 °C, implied hereafter), and this was controlled through constant aeration at 6 LPM (atmospheric air only) with a mixing cascade operating from 350–1200 rpm using a single six-blade Rushton turbine measuring six centimeters in diameter. After the mixing cascade could no longer maintain DO, the cultivation was continued for oxygen limitation-induced PHA accumulation with the DO probe reading 0% to 3% air saturation.

Feeding of octanoic acid and a 200 g L^−1^ solution of (NH_4_)_2_SO_4_ was accomplished through two 10 mL precision injector syringes (Hamilton Company, Reno, NV, USA) coupled to three-way solenoid valves (Omnifit, Biochem Fluidics, Boonton, NJ, USA). The syringes were driven by pulse motors (SmartMotor^TM^, Moog Animatics, Mountain View, CA, USA) and automated by LabBoss software (Scion, Rotorua, New Zealand). 

### 2.3. Feeding Strategies

The above-described apparatus was used to feed pulses of octanoic acid in small amounts (2.5 to 9.5 mL at a time, total concentrations of approximately 5 to 20 mM) along with sufficient (NH_4_)_2_SO_4_ for balanced growth until 20 hours (h), after which (NH_4_)_2_SO_4_ was no longer fed. Pulse feeding was conducted in response to either a drop in the off-gas CO_2_ concentration or a sudden rise in the DO, which indicated depletion of the carbon source. The volume of the pulse depended on the phase of growth, generally with smaller pulses (5 mM) in the early stages of growth or in late stationary phase. Experiments were generally completed by 27 h post inoculation and were terminated when: (1) further addition of carbon had no effect on the (already declining) CO_2_ production rates; (2) the measured OD_600_ decreased despite the presence of excess carbon; or (3) excessive and uncontrollable foaming, which usually occurred after the onset of the two previous symptoms. These experiments were replicated three times. 

The pulse feed process was subsequently modeled and run as a continuous drip feed process (i.e., proactive feeding at predetermined rate) in order to compare with the pulse feed (i.e., a reactive feeding) approach. In the continuous approach, the motors driving the injector syringes were run continuously at very low speed, such that feeding was continual drop-wise addition as opposed to injecting a slug of octanoic acid at one time. From the pulse feed data, curves for the total uptake of carbon and NH_4_ were plotted over time (*C(t)* and *N(t)*, respectively). A satisfactory fit could be obtained with a piecewise function, in which the growth phase was fitted with an exponential function, whereas the PHA accumulation phase was fitted with a quadratic function. This trend is in agreement with what has been previously reported by MacLean et al. [18]. These equations were programmed into LabBoss software to automate a continuous drip feed process and compare the results to the pulse feed process. Within the 27 h cultivation, a total of 185 mL octanoic acid and 235 mL of the 200 g L^−1^ (NH_4_)_2_SO_4_ solution were fed. These experiments were repeated three times.

(1) $$C\left(t\right)=\{\begin{array}{cc} 0.273e^{0.366t}, & t\leq 10\;h\\ -0.0293t^{2}+3.478t-2.157, & t>10\;h \end{array}.$$

(2) $$N\left(t\right)=\{\begin{array}{cc} 0.034e^{0.418t}, & t\leq 9\;h\\ -0.0151t^{2}+0.628t-3.0112, & t>9\;h\\ 0,\; & t>20\;h \end{array}.$$

### 2.4. Measurement of CO_2_ and Mass Balancing

The CO_2_ signal was measured at a mass-to-charge (*m/z*) of 44 using a Hiden HPR-40 dissolved species membrane-inlet mass spectrometer (Hiden Analytical, Warrington, UK). Total CO_2_ was quantified by integrating the measured off-gas concentration over the airflow rate to the bioreactor, while also accounting for dissolved carbonate species as described previously [37]. It was found that the off-gas CO_2_ concentration was a more rapid indicator of carbon depletion during growth, while during PHA accumulation, changes in the off-gas CO_2_ concentration were not as pronounced, making the DO signal a more reliable indicator. The carbon balance was performed assuming that all consumed carbon could be accounted for through measured CO_2_, PHA biomass (*X_PHA_*), and non-PHA residual cell mass (*X_r_*). The mass balance followed the same approach described previously [26,27].

### 2.5. Yield Coefficients

The yield coefficient of *X_r_* from NH_4_ (*Y_Xr/N_*) was determined to be 6.1 g g^−1^ from previous work [27]. This value is in reasonable agreement with the *Y_Xr/N_* of 5.44 g g^−1^ reported by Sun et al. [29]. Similarly, the yield coefficients of *X_r_* and PHA from octanoic acid (*Y_Xr/S_* and *Y_PHA/S_*, respectively) were derived from previous data as 0.72 g g^−1^ and 0.62 g g^−1^. The *Y_PHA/S_* value was similar to the 0.63 g g^−1^ reported previously for nonanoic acid [34]. The *Y_Xr/S_* measured in this work is similar to the growth-phase *Y_X/S_* of 0.8 g g^−1^ reported previously for growth on nonanoic acid, although it is not clear if this value considered total biomass (*X_t_*) or *X_r_* only. Other medium components (PO_4_^3−^, Mg^2+^, Fe^3+^) were assumed to be in excess on the basis of the amount added to the medium and the yield coefficients reported previously [29,38]. The validity of this assumption was checked through measurement of residual trace metals in the culture supernatant (described below). 

### 2.6. Sample Treatment

Samples (20 to 40 mL) were periodically withdrawn from the bioreactor, generally in 1 to 3 h intervals. These were centrifuged for 10 minutes at 12,500× *g*. The pellet was washed once in PBS buffer, transferred into a pre-weighed 20 mL aluminum tray and dried at 60 °C until no further loss of mass was detected to determine [*X_t_*] (g L^−1^ cell dry mass, CDM). The PHA was extracted in chloroform using the acid-catalyzed methanolysis procedure [39]. The PHA content (*%_PHA_*) of the biomass was quantified using a gas chromatograph equipped with a flame ionization detector (GC-FID) identical to that described previously [40]. The supernatant was decanted and stored at −20 °C until further analyses could be performed. These are described below. 

### 2.7. Measurement of Residual Carbon, NH_4_-N PO_4_^3−^-P, and Trace Medium Components

Residual octanoic acid in the culture supernatants was measured by GC-FID as previously described [26]. Residual free NH_4_ was measured spectrophotometrically at 630 nm via the indophenol blue method (Lachat QuikChem^®^ Method 10–107-06-1-J) as previously described [27]. Residual PO_4_^3−^ was also measured spectrophotometrically in a flow injection system using the Quikchem^®^ method 10-115-01-1-A. Briefly, PO_4_^3−^ reacts with ammonium molybdate and antimony tartarate to form a complex that produced a blue color when reduced with ascorbic acid, which is measured at 880 nm. Samples were diluted 250X prior to measurement.

The concentration of trace elements was measured via inductively coupled plasma optical emission spectrophotometer (ICP-OES) at intervals over time. Samples were prepared without dilution, although the samples were acidified by adding nitric acid to a final concentration of 2% (vol vol^−1^) prior to filtering through a 0.2 μm filter. The eluent used for the ICP-OES was also 2% nitric acid. 

## 3. Results 

### 3.1. Pulse Feed Strategy 

**Figure 1:**
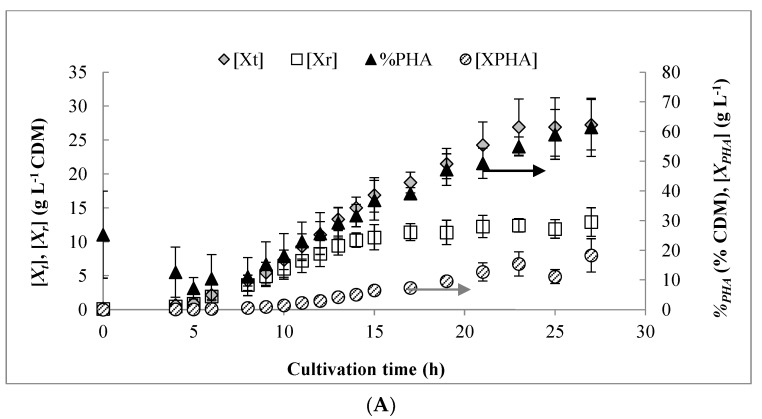

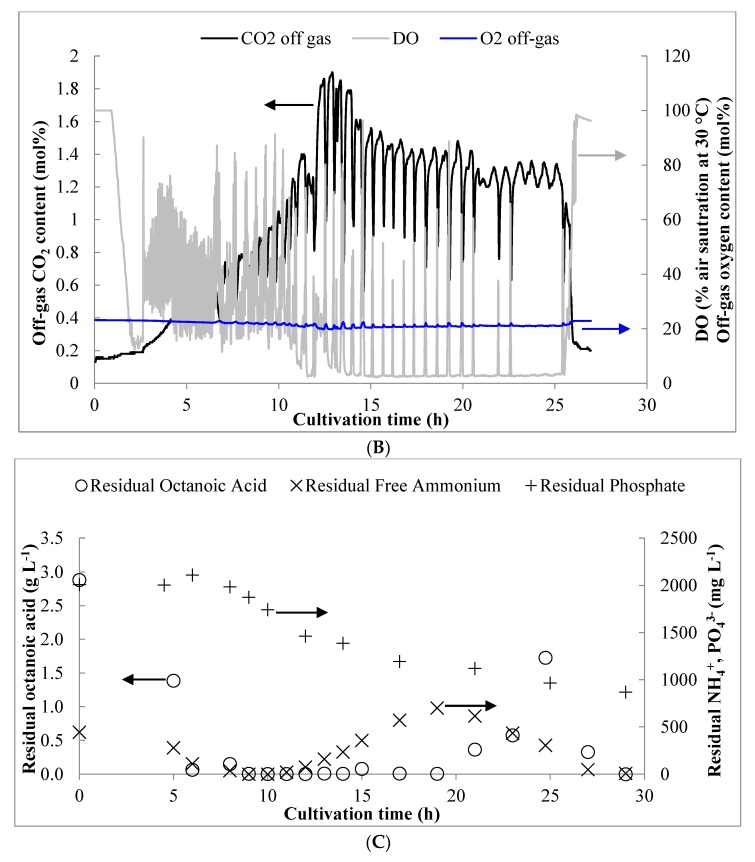
(**A**) Results for biomass and polyhydroxyalkanoate (PHA) production obtained over the course of the pulse feed experiments. (**B**) Representative profile for dissolved oxygen (DO) content over time as well as off-gas CO_2_ and O_2_ content. (**C**) Residual concentrations of octanoic acid, free ammonium, and phosphate observed during the bench scale pulse feed experiments. Results shown are for a representative pulse feed experiment. Error bars represent standard deviations between three biological replicates.

Figure 1A shows the results for biomass and PHA production obtained from three independent pulse fed-batch experiments. Despite constant aeration set at 6 LPM and a cascading stirrer reaching its maximal value, the DO could not be maintained at the intended set point of 40% beyond 11 h (Figure 1B). By 14 h, the DO was consistently below 5% while excess carbon was present. From previous results, these conditions are normally expected to cause a shift in carbon flux from growth to PHA synthesis [27]. However, the PHA content of the biomass started increasing significantly from about 8 h onward, while DO was still maintained at relatively high levels. The low residual levels of carbon and/or NH_4_ detected in the culture medium at this time (discussed below) may have slowed the growth rate and caused storage of carbon as PHA [34]. This is supported by the observation that *X_r_* production continued until 14 h and then declined to nearly negligible values due to DO limitation, which is consistent with previous work using octanoic acid [26]. 

### 3.2. Residual Concentrations of Octanoic Acid, NH_4_^+^-N, and PO_4_^3−^-P

Figure 1C shows the profiles of residual carbon NH_4_, and PO_4_^3−^ detected in the culture medium. As shown, the initial 20 mM of carbon added to the reactor was depleted within 6 h. Subsequently, octanoic acid was fed in small, frequent pulses (~5 to 20 mM) in response to either a drop in off-gas CO_2_ concentration or a rise in DO signal, and the residual concentration remained low until after 20 h, at which point slight excess was observed at the indicated sampling points. NH_4_ appeared to have been briefly limited around 9–10 h, but began to accumulate after 10 h, likely due to cessation of growth from the onset of O_2_-limitation. By 20 h, the residual NH_4_ concentration reached 700 mg L^−1^ and feeding of (NH_4_)_2_SO_4_ was stopped (to prevent accumulation to toxic levels). The excess was consumed to a final concentration of 49 mg L^−1^ by 27 h, resulting in an uptake rate of 5.90 ± 0.25 mg NH_4_ g *X_r_*^−1^ h^−1^. By comparison, the measured uptake rate of NH_4_ during the growth phase (0–14 h) was 286.5 ± 25.9 mg NH_4_ g *X_r_*^−1^ h^−1^.

Phosphate was consumed most rapidly during the growth phase from 6 to 12 h and the slope of a plot between PO_4_^3−^ consumption and *X_r_* production (*R^2^* = 0.91) produced a yield coefficient of 13.48 g *X_r_* g PO_4_^3− −1^ (Table 1). This is similar to the value of 13.7 g g^−1^ reported by Sun et al. [29] for *P. putida* KT2440. Consumption of PO_4_^3−^ was also observed during the PHA accumulation phase (14 h and onward) at the rate of 3.08 ± 0.96 mg PO_4_^3−^ g *X_r_*^−1^ h^−1^. Prior to 14 h, the obtained growth-phase PO_4_ uptake rate was 52.7 ± 11.1 mg PO_4_ g *X_r_*^−1^ h^−1^.

### 3.3. Residual Trace Elements

**Figure 2:**
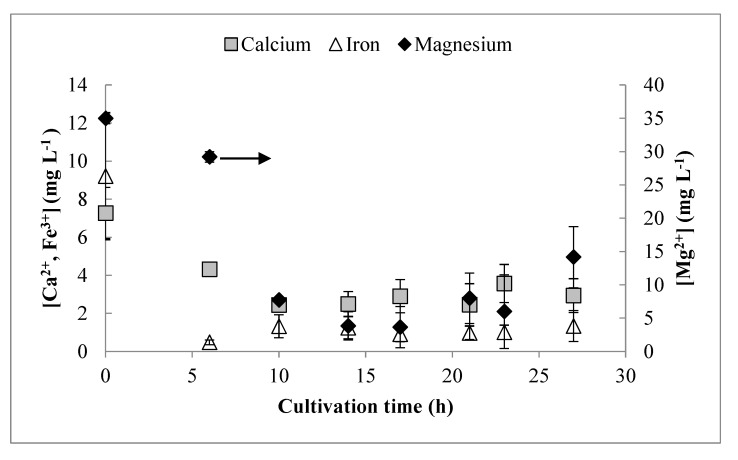
Analysis of residual concentrations of trace elements (calcium, iron, magnesium) in the culture supernatant by inductively coupled plasma optical emission spectrophotometer (ICP-OES). Error bars represent standard deviations between three biological replicates.

For measurement of trace residual metals with ICP-OES, satisfactory resolution was found for Fe^3+^, Ca^2+^, and Mg^2+^ (Figure 2). The concentrations of Mg^2+^ decreased until 14 h, and then the concentration began to slowly increase. The concentration of Fe^3+^ decreased rapidly until 6 h, and then remained relatively constant at a low concentration for the remainder of the cultivation. The concentration of Ca^2+^ decreased until 10 h and showed little change subsequently. Poor resolution was obtained for Cu^2+^, Mn^2+^, and Zn^2+^. Even initially, these were present in extremely low concentrations, but did not appear to be depleted at any point during the cultivation. Growth-phase yield coefficients (obtained prior to 12 h) for all detectable medium components are shown in Table 1. None of these trace metals reported zero concentration values at any time and, therefore, were likely not limiting. However, for cultivations targeting even higher cell densities, increasing the concentration of these metals would be advisable, particularly for Fe^3+^ and Mg^2+^. 

### 3.4. Modeling of Feeding Rates—Continuous Drip Feed Strategy

**Figure 3:**
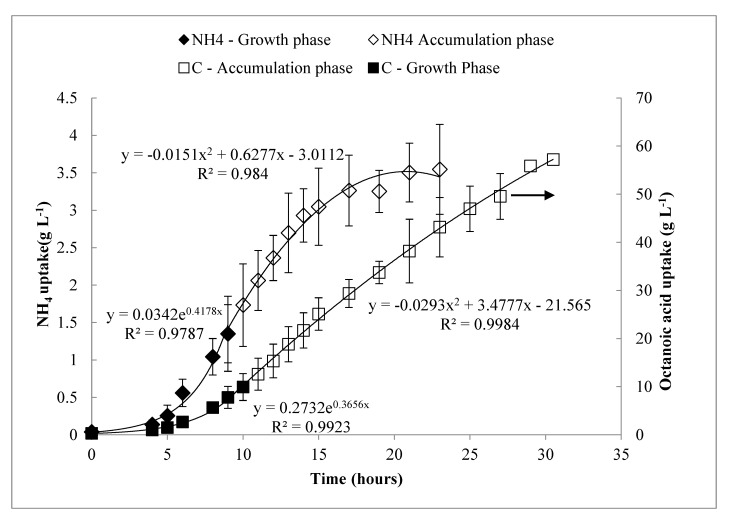
Modeled cumulative feeding of carbon and ammonium over the time course of the pulse feed experiments. Error bars represent standard deviations between three biological replicates.

**Figure 4:**
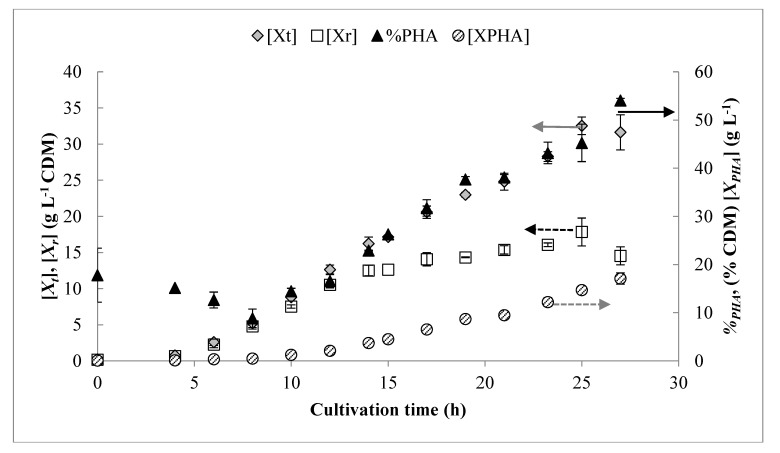
Growth curves shown for continuous drip strategy derived from modeled feeding rates. Error bars represent standard deviations between three biological replicates.

Curves for carbon and ammonium uptake rates over time in the pulse feed process were modeled as piecewise functions, shown in Figure 3. The results from three replicates of these experiments are shown in Figure 4 and summarized in Table 2 with comparison to previous work. The process was very consistent between replicates, and the concentration profiles for DO, carbon, and NH_4_ behaved similarly to the pulse feed experiments described above. As shown in Table 2, the drip feed strategy appeared to favor production of *X_r_* over *X_PHA_*, as indicated by the slightly higher [*X_t_*], lower *%_PHA_*, lower *Y_PHA/S_*, and a lower rate of PHA synthesis (indicated by specific productivity, *Q_s_*) during the accumulation phase. 

### 3.5. PHA Composition

The detected monomer composition for both fed-batch cultivation processes is shown in Table 3. The monomeric composition was similar in both cases, but the dominant monomer was C8 (90–93 mol%), followed by C6 (6–7 mol%), with traces of C10 and C12. 

### 3.6. Carbon Flux and Yield Analysis

**Figure 5:**
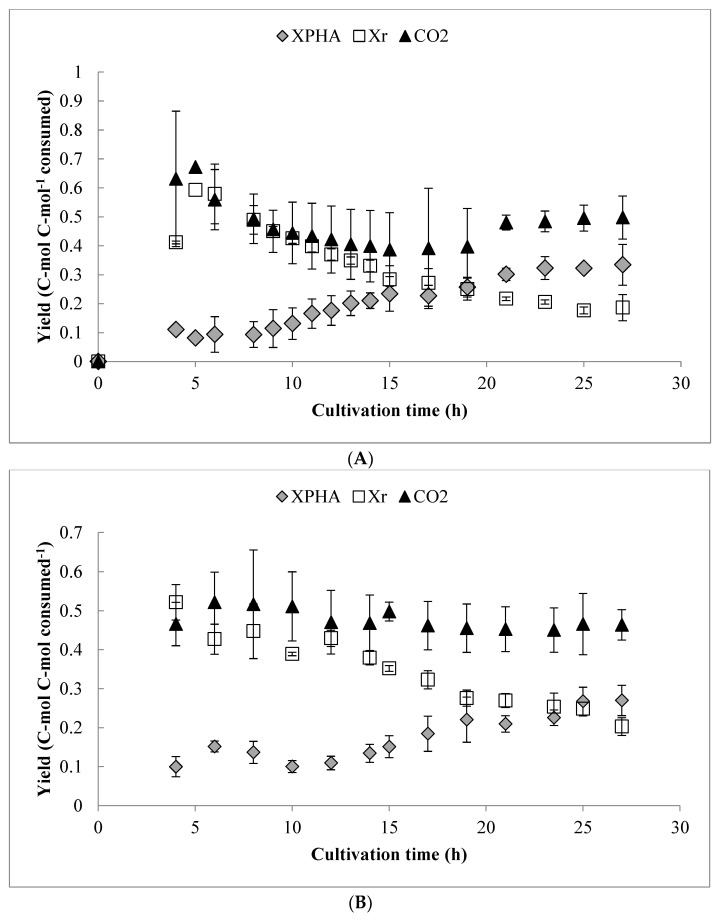
Overall (cumulative) yield analysis for *X_PHA_*, *X_r_*, and CO_2_ over the duration of: (**A**) the pulse fed-batch cultivations and (**B**) the continuous drip feed experiments. Values expressed on a C-mol basis. Error bars represent standard deviations between three biological replicates.

An overall yield analysis (on a C-mol basis) is shown in Figure 5, for both pulse-fed and continuous drip feed experiments. In either case, there was little change in the CO_2_ production (which accounted for approximately 50% of the consumed carbon on a molar basis) throughout the cultivation, which is consistent with previous results [26,27]. However, the carbon flux to *X_r_* was higher during the O_2_-limited phase in comparison with previous work, which caused lower PHA yield. This comparison is also shown in Table 2, along with several other key indicators of process performance. The maximum observed *Q_s_* and overall *Y_PHA/S_* yield from octanoic acid were similar in both cases, while the maximum *Y_PHA/S_* during the O_2_-limited PHA storage phase was somewhat lower than in previous batch experiments. The carbon recovery approached one (0.9 ± 0.15, Table 2) for the fed-batch experiments, but was generally more variable than in previous batch experiments. Worth noting is that, as shown in Table 2, if only carbon consumed during the PHA storage phase is considered, a significantly higher proportion was allocated to PHA synthesis in the pulse feed experiments compared to the drip feed experiments. 

## 4. Discussion

This work has shown that: (1) oxygen limitation can be a viable strategy for inducing mcl-PHA accumulation from fatty acids using *P. putida* in HCD fed-batch cultivations; and (2) this process can be rapidly optimized through simple pulse feeding in response to DO and/or off-gas CO_2_ signals as real-time feedback variables. One advantage of this approach is that it eliminates the need to predict growth rates or feeding rates over time, which can lead to complicated automation and programming [29]. Previously, a similar strategy proved to be a significant challenge to optimize when using toxic substrates like octanoic acid [26]. Furthermore, this approach can be implemented with standard bioreactor equipment (i.e., the setup could be as simple as a DO probe and a feed pump), and should be independent of the bioreactor type, configuration, and to some extent also the scale and mass transfer capability. Although O_2_-limitation has been reported to cause excessive foaming and termination of fed-batch processes [17,34,41], in this work we found that foam was manageable throughout the O_2_-limited phase (except for at the very end of the process). In fact, foaming was observed upon carbon limitation in the pulse feed process, and was alleviated by further addition of octanoic acid. 

During the storage phase, consumption of both NH_4_ and PO_4_ was observed, albeit at a considerably slower rate than during growth. This may be due to residual growth and/or maintenance functions during the O_2_-limited PHA storage phase, which could be advantageous compared to strict N or P limitation. Andin et al. [42] examined the effect of maintaining a low residual growth rate on metabolic flux during synthesis of mcl-PHA from fatty acids in fed-batch culture using *P. putida* KT2440. Their analysis showed that when residual growth was present, the reduced co-factors from mcl-PHA synthesis (NADH, FADH_2_) could be coupled to anabolic demand, and this improved the overall mcl-PHA yield from approximately 0.6 to 0.7 C-mol C-mol^−1^. Another study reported a similar finding for PHB synthesized from butyrate using *C. necator* [43]. In this work, the yield coefficient during the storage phase was lower at 0.52 ± 0.13 C-mol C-mol^−1^ and the average residual growth rate during the storage phase was 0.03 ± 0.01 h^−1^ (Table 2). As suggested previously, co-feeding LCFAs may help improve residual growth during the PHA storage phase, and perhaps even further improve overall *Y_PHA/S_* [26].

Differences in the carbon feeding methods were also evaluated via a continuous drip feed process developed using an empirical model of the pulse feed data. Although the processes were largely similar, more favorable PHA storage characteristics (higher *Q_s, max_* and *Y_PHA/S_*) were observed under pulse feeding conditions, whereas the drip feed process resulted in significantly higher [*X_r_*], but lower *Y_PHA/S_* and *Q_s, max_*. 

A previous study indicated a significant improvement in *%_PHA_* (from 25% to 44% of CDM) when *Pseudomonas* sp. G101 was pulse-fed waste canola oil, in comparison to a continuous drip feed [44]. The results of the present study do not suggest such a drastic difference between drip feed and pulse feed methodologies. However, the findings do imply that pulse feeding may help to maximize the yield and synthesis rate of mcl-PHA during the accumulation phase, whereas a continuous drip feed process may help boost biomass production during the growth phase. This may be due to the constant cycling between carbon excess and carbon-limited conditions imposed by the pulse feed strategy. However, it should be noted that during the course of these drip feed experiments, periodic carbon limitation was still imposed when the injector syringe was briefly paused to refill, which may have dampened the observed effect. Perhaps a more optimized strategy could result from coupling a continuous, drip feed strategy for growth with a pulse feed strategy in the PHA accumulation phase. 

The effects of the feeding strategy on molecular weight of the polymer is an issue that needs to be addressed in mcl-PHA production when considering different feeding strategies, and should be the subject of future studies. Indeed, it has been previously shown that molecular mass of the polymer can be affected by cultivation conditions, even for PHB homopolymers [45,46]. Further, understanding the effects of the feeding strategy on polymer synthesis (constant carbon limitation compared to periodic carbon limitation) at a more mechanistic level (polymer chain termination, preferential back consumption of lower molecular weight polymers) would be a valuable contribution [4].

Interestingly, mcl-PHA synthesis characteristics were not improved over previous batch [27] and/or simple fed-batch experiments [26]. This difference might reveal that the kinetics of mcl-PHA synthesis and storage are perhaps more well suited to the simpler, more gradually changing chemical environment of a batch system. This implies that the main mode of advancing HCD fed-batch productivities, at least in this work, was solely through boosting [*X_t_*] rather than improving mcl-PHA synthesis characteristics. The overall carbon recovery was also somewhat lower in the HCD fed-batch cultivations, which could indicate that carbon was used for end products other than *X_PHA_*, *X_r_*, and CO_2_. These were not accounted for in this work.

Compared to other HCD fed-batch processes, several previous bench-scale studies have demonstrated improved results over the fed-batch process described in this work [17,18,33,34]. Many of these studies have used an aeration medium with enriched oxygen content, which increases the driving force for oxygen transfer nearly fivefold, and has been shown to improve PHA productivity nearly fourfold in bench-scale reactors [47]. Thus, using a technique to increase the driving force for oxygen transfer (enriched O_2_, bioreactor pressurization) would help make this fed-batch process more competitive with previous studies documented in the literature, but that was not within the objectives of this work. It is important to point out that the main contribution of this work was to show the effects of low-DO environments in HCD fed-batch cultivations under different feeding strategies, and not necessarily to maximize overall *Q_v_* using techniques to keep DO in excess. Although those are worthwhile pursuits, they have been extensively explored in the literature, but have not necessarily been replicated in larger scale bioreactors [22]. Regardless of how much the oxygen transfer rate might be improved, at some point microaerophilic conditions will persist beyond a certain cell density. 

## 5. Conclusions

A fed-batch method was developed using a CO_2_ and/or DO-based pulse feed strategy using atmospheric air as the aeration medium and O_2_-limitation as the main driving force for mcl-PHA production. The process consistently produced 25–30 g L^−1^ total biomass and resulted in an overall *Q_v_* of 0.66 ± 0.14 g L^−1^ h^−1^. Interestingly, no improvement to mcl-PHA synthesis characteristics (*Q_s_*, *Y_PHA/S_*) was observed when compared to previous oxygen-limited batch cultivations, meaning that the productivity advances were solely due to increased total biomass. Furthermore, the overall carbon recovery was lower, suggesting that carbon may be utilized less efficiently in HCD cultures. Finally, while a continuous drip feed strategy favored growth and production of *X_r_*, a pulse feed strategy favored the production of mcl-PHA during the storage phase.

## Figures and Tables

**Table 1:** Yield coefficients for C, N, P, and certain (detectable) trace elements.

Medium Component	Yield Coefficient
Octanoic Acid (g g^−1^)	0.62 ^a^
NH_4_^+^ (g g^−1^)	6.1 ^a^
PO_4_^3−^ (g g^−1^)	13.5
Ca^2+^ (g mg^−1^)	2.6
Cu^2+^ (g mg^−1^)	15.5
Fe^3+^ (g mg^−1^)	2.2
Mg^2+^ (g mg^−1^)	0.5

^a^ results obtained from previous batch tests, Blunt et al. [27].

**Table 2:** Comparison of key process performance indicators of previous batch cultivations with the current fed-batch process.

Process Performance Indicator	Previous Batch Results ^a^	Pulse Feed Strategy (at 27 h Unless Otherwise Stated)	Continuous, Drip Feed Strategy (at 27 h Unless Otherwise Stated)
[*X_t_*] (g L^−1^)	2.37 ± 0.1	28.9 ± 4.0	32.4 ± 0.9
*%_PHA_* (g g^−1^)	44.4 ± 1.3	60.6 ± 8.2	52.9 ± 2.5
[*X_r_*] (g L^−1^)	2.37 ± 0.5	11.2 ± 1.5	17.4 ± 2.1 ^b^
[*X_PHA_*] (g L^−1^)	1.01 ± 0.12	17.7 ± 4.8	15.4 ± 1.2
μ_avg/Xr_, growth phase (h^−1^)	0.29 ± 0.03	0.35 ± 0.11 (0–14 h)	0.31 ± 0.03 (0–14 h)
μ_avg/Xr_, storage phase (h^−1^)	0.11 ± 0.01	0.03 ± 0.01 (14–27 h)	0.03 ± 0.01 (14–27 h)
*Q_v, final_* (g L^−1^ h^−1^)	0.08 ± 0.00	0.61 ± 0.12	0.60 ± 0.04
*Q_v_, _max_* (g L^−1^ h^−1^)	0.08 ± 0.01	0.66 ± 0.14 (23–27 h)	0.60 ± 0.04 (27 h)
*Q_s, max_* (g PHA g *X_r_*^−1^ h^−1^)	0.18 ± 0.03	0.18 ± 0.03 (15–19 h)	0.10 ± 0.03 (23–25 h) ^b^
*Q_s, avg_* (g PHA g *X_r_*^−1^ h^−1^)	0.11 ± 0.00	0.09 ± 0.01 (14–27 h)	0.06 ± 0.01 (14–27 h) ^b^
*Y_PHA/S_*, overall (C-mol C-mol^−1^)	0.35 ± 0.04	0.33 ± 0.05	0.26 ± 0.04
*Y_PHA/S_*, storage phase (C-mol C-mol^−1^)	0.57 ± 0.05	0.52 ± 0.13 (14–27 h)	0.31 ± 0.06 (14–27 h) ^b^
Carbon Recovery	1.04 ± 0.00	0.90 ± 0.15	0.89 ± 0.04

^a^ results chosen for O_2_-limited conditions with 6 LPM aeration and 250 rpm mixing (*k_L_a* = 78 h^−1^), obtained from Blunt et al. [27]; ^b^ results that are compared for the two fed-batch strategies that are statistically different using a two-tailed homoscedastic comparison of sample means (*p* < 0.05). Tolerances indicate standard deviations between three biological replicates.

**Table 3:** Monomer composition of polymer synthesized from octanoic acid using *P. putida* LS46 under different fed-batch strategies. Tolerances indicate standard deviations between three biological replicates.

Feeding Strategy	C6	C8	C10	C12
	(Mol %)			
Pulse	5.8 ± 0.6	92.9 ± 0.6	1.1 ± 0.2	0.6 ± 0.2
Continuous drip	7.4 ± 0.2	89.7± 0.3	2.2 ± 0.1	0.6 ± 0.2

## Abbreviations

CDM	cell dry mass (g L^−1^)
*C(t)*	cumulative octanoic acid uptake as a function of time (g L^−1^)
*N(t)*	cumulative (NH_4_)_2_SO_4_ uptake as a function of time (g L^−1^)
*Qv*	overall volumetric productivity of PHA (g L^−1^ h^−1^)
*Qs*	specific productivity or PHA synthesis rate (g g *X_r_*^−1^ h^−1^)
*X_PHA_*	PHA biomass (g)
*X_r_*	non-PHA (residual) cell mass (g)
*X_t_*	total biomass (g)
*Y_PHA/S_*	yield coefficient of PHA per unit carbon substrate consumed (g g^−1^ or C-mol C-mol^−1^)
*Y_Xr/N_*	yield coefficient of non-PHA cell mass per unit ammonium consumed (g g^−1^ or mol mol^−1^)
*Y_Xr/S_*	yield coefficient of non-PHA cell mass per unit carbon substrate consumed (g g^−1^ or C-mol C-mol^−1^)
*Y_X/S_*	yield coefficient of total biomass per unit carbon substrate consumed (g g^−1^ or mol mol^−1^)
μ*_avg/Xr_*	average specific growth rate over a defined period (h^−1^, calculated using increases in *X_r_*)
*%_PHA_*	intracellular PHA content (% of CDM)
[ ]	concentration braces (g L^−1^ or mg L^−1^)
